# Investigating the Youth Sports Development Pathway Within a South African Context

**DOI:** 10.3389/fpsyg.2021.694548

**Published:** 2021-07-26

**Authors:** Liandi van den Berg, Petronella Jonck, Jhalukpreya Surujlal

**Affiliations:** ^1^TELIT-SA, Faculty of Economic and Management Sciences, North-West University, Vanderbijlpark, South Africa; ^2^GIFT, Faculty of Economic and Management Sciences, North-West University, Mahikeng, South Africa; ^3^WorkWell, Faculty of Economic and Management Sciences, North-West University, Potchefstroom, South Africa

**Keywords:** athlete, development, support, pathways, talent

## Abstract

The past two decades witnessed increased participation in professional as well as amateur sport, giving impetus to concomitant amplified interest in long-term athlete development (LTAD). LTAD has been described as the structured and progressive growth of an athlete through different stages of development resulting in some athletes achieving elite sport status. Furthermore, the interest in athletic career development from a holistic perspective has contributed to management approaches underscoring sustainable talent development and participation in sport. The current study investigated youth sports development pathways through both models of development within a South African context. A descriptive quantitative cross-sectional design was used to generate a convenient sample of athletes (*N* = 267). The Talent Development Environment Questionnaire (TDEQ) was administered, which in previous studies produced acceptable psychometric properties. Principal factor analysis, confirmatory factor analysis, Monte Carlo parallel simulation, MANOVA, and hierarchical regression were performed to analyze the data. The TDEQ was validated for the South African context and was found to measure four components, namely *supportive and challenging environment*, *development fundamentals*, *support networks*, and *long-term development*. Respondents in the various developmental categories of novice, advanced and elite student-athletes were not statistically significantly influenced by any of the four factors. Controlling for the talent developmental phase, the model proposed did not statistically significantly predict the development pathway of youth athletes. The results provide evidence with some practical significance as supportive and challenging environment and long-term development focus reported a small effect. Further research is warranted to develop a more suitable measuring instrument to measure the talent development pathway within the investigated athlete environment.

## Introduction

Internationally, early talent identification and development has become increasingly important within the context of youth sport structures and programs. In the quest to achieve international and elite levels of competition, national sport systems provide a blueprint for youth athlete development (Hollings, 2013). Burnett (2017) noted that national policy frameworks and promulgated documents guide and align stakeholders in a delivering system or pipeline to foster athlete development. Provincial and national development programs focus on managing practices that influence athletes’ holistic development, with the purpose of positioning the aforementioned on a path of elite sport performance from an early age (Hardell, 2017).

Sporting talent development is a long-term process during which young athletes progress through various stages with committed practice and training required (Balyi et al., 2013). The various stages of athletic progression have universally been classified as a systematic and scientific approach, which resulted increasingly in uniform models of development pathways established and implemented for athletes (Trofimenko et al., 2019). A prominent model that has influenced sport development programs significantly is the long-term athlete development (LTAD) model, which proposes a systematic and progressive longitudinal program for athlete development (Balyi et al., 2013). The LTAD takes into consideration the maturational status of individual athletes and differentiate between early and late specialization (Balyi and Hamilton, 2010). Early specialization requires a four-phase model, while late specialization requires a six-stage model (Balyi and Hamilton, 2010). The two different LTAD models indicate a varied approach to the development of expertise depending on the sport (Balyi and Hamilton, 2010; Hardell, 2017). In addition, numerous factors such as resources, coaches’ provision, and coach development, training facilities, financial aid, athletic support and opportunity to compete, influence talent development over an extended period (De Bosscher et al., 2013). The LTAD model is one of the theoretical frameworks put forward during the past few decades aiming to foster athlete expertise developed over time (Coutinho et al., 2016). While the LTAD model conceptualizes athlete development from a *talent development* perspective, other theoretical frameworks emerged focusing on a *career transition* stance (Bruner et al., 2010). Career transition models differ from talent development models since the first mentioned describe factors, demands, coping processes and consequences of transition periods compared to development models, which focus on specific influential factors needed during a developmental stage (Coutinho et al., 2016). Consequently, the LTAD model was developed from a talent development perspective, while the transitioning perspective advanced a holistic view on athletic career development (Wing Hong To et al., 2013; Wylleman et al., 2013). A dearth of research emphasizes both perspectives simultaneously, therefore this study incorporated both models of athlete developmental pathways to investigate the combined elements that describe South African student-athletes’ pathways. The framework of investigation related to overlapping elements within both models, and assisted the researchers to build a cohesive body of knowledge on student-athletes’ development pathways evaluated within the South African sport context. Against the stated background the aim of the study was to investigate the sports development pathway of student-athletes in terms of four aspects, namely supportive and challenging environment, development fundamentals, support network and long-term development focus, as measured by the Talent Development Environment Questionnaire (TDEQ).

## Literature Review

**FIGURE 1:**
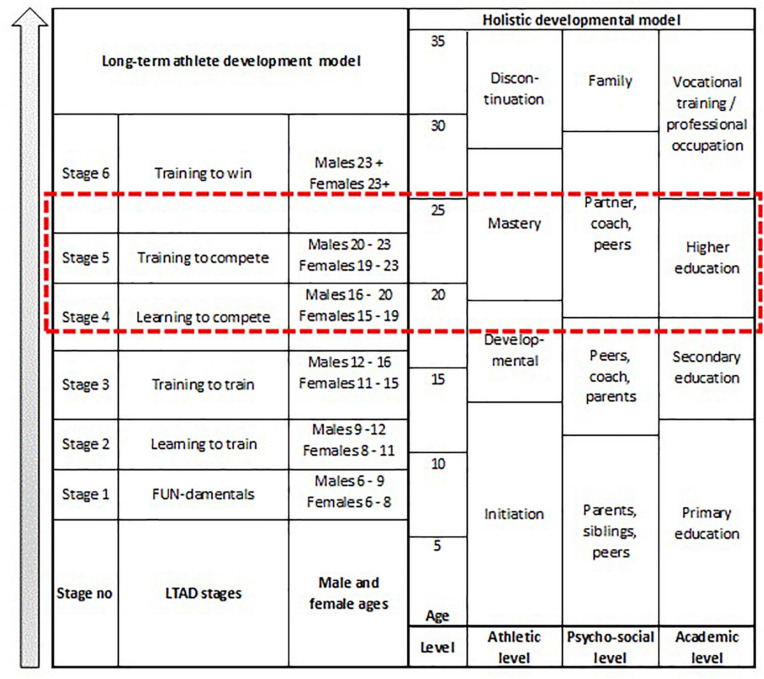
Combined models of LTAD (Balyi et al., 2013) and holistic perspective development (Wylleman and Lavallee, 2004).

The long-term sport talent development process is complex, and can be explained by two primary perspectives focusing on *talent development* and *career transitioning*, respectively. The afore stated frameworks aim to conceptualize long-term athlete talent development, integrating specific factors necessary during each stage and transition to progress through the sporting ranks and achieve success as junior and senior athletes (De Bosscher et al., 2013). Additionally, the primary perspective’s purpose is to increasingly define the talent development pathway followed by successful athletes (Balyi et al., 2013), to understand the factors that influence high performance sport, and to address it throughout athletes’ developmental pathway (Coutinho et al., 2016). Figure 1 compares the LTAD and holistic development models, and provide a framework of investigation regarding comparative elements within the two models. The framework provides a visual comparison of the stages, ages and levels within the models of Balyi et al. (2013) and Wylleman and Lavallee (2004), as indicated by the red dotted rectangle.

### Long-Term Athlete Development Model

The LTAD model was created to improve the quality of sport programs, since successful sport participation requires planned and systematic progressive development of individual athletes (Ellerton, 2019). The LTAD provides guidance to coaches, administrators, parents, and sport scientists regarding sport development stages and pathways, where developmentally appropriate training and competition programs at specific ages should be in place (Wing Hong To et al., 2013; Ellerton, 2019). The LTAD, *inter alia*, suggests a varied approach to talent development and refers to early specialization and early diversification (or late specialization) for the talent development pathway of athletes (Balyi et al., 2013; Coutinho et al., 2016). For the sake of clarity, specialization refers to athletes limiting their participation to a single sport to train and compete year-round (Ellerton, 2019). Early specialization refers to the investment in one sport with deliberate practice from a young age, whereas late specialization involves early participation in a wide range of sports (Coutinho et al., 2016). Early talent identification, selection and specialization are more prevalently ascribed to increased youth sport commercialization (Eckstein, 2019). Likewise, parents, coaches, clubs, and schools consider the pathway to elite levels of competition through early and single sport specialization (Malina, 2010). In this regard, youth athletes spend many training hours under the watchful eye of skilled coaches in deliberate practice (Ericsson et al., 1993). The hours spent training with coaches not only purpose to develop skills to compete at junior elite levels, but also to advance to higher competitive levels at earlier ages and obtain university scholarships at a later stage (Malina, 2010; Hardell, 2017). However, since research supports that early sport specialization has resulted in early dropout and is not essential for exceptional sport performance as an adult, diversified approaches to sport development have been pursued by various sport stakeholders (Baker, 2003; Côté et al., 2003). Likewise, only a few sports are categorized as early specialization sports (for example acrobatic and artistic sports such as gymnastics and figure skating), and therefore many countries’ NGBs adopted the late specialization LTAD model as an integrated and collaborative approach for athletes to develop from a young age up to elite level (Ellerton, 2019; Nolte and Hollander, 2020).

The six-step process of the late specialization pathway is divided into the following stages: fundamental (males 6 to 9 years/females 6 to 8 years), learning to train (males 9 to 12 year/females 8 to 11 years), training to train (males 12 to 16 years/females 11 to 15 years), learning to compete (males 16 to 20/females 15 to 19), training to compete (males 20 to 23 years/females 19 to 23 years) and training to win (males and females 23 years and older) (Balyi et al., 2013). The six-step late specialization model posits that athletes need to diversify sport participation and systematically train over a period of time in numerous different activities. Evidence also suggests that athletes following the late specialization approach were not at a disadvantage to proceed to elite levels of participation compared to athletes who specialized much earlier (Côté et al., 2003). The late specializing approach therefore implies that athletes could progress through the different LTAD stages as they get older and at the age of 16 years (males) and 15 years (females), be proficient in a sport with basic skills after which they begin to learn to compete and become more competitive at higher levels within a specific sport (Balyi and Hamilton, 2010; Ellerton, 2019). The learning to compete stage (during the secondary educational phase) resolves to optimize fitness, prepare sport-, individual- and position-specific skills and expose athletes to higher levels of junior competitions. Athletes participate in year-round specific and high-level training, where the focus is on optimum preparation, physical and mental development as well as individual adaptation to the demanding training and competitive environment (Balyi and Hamilton, 2010). When males reach the age of 18 and females 17 years, according to the LTAD, there should be a high level of skill proficiency because of two to three years of exposure to high quality training and/or elite competition levels. The transition from learning to compete and training to compete stages, coincides with a career transition period that athletes experience when they move from secondary to tertiary education. In this regard, when athletes leave high school and enter tertiary education, they transition into senior sport and have to successfully negotiate complex and unique demands and enter the LTAD training to win stage (Vallée and Bloom, 2005; Balyi and Hamilton, 2010; Hollings, 2013). It is pivotal to consider the career transitioning stages and factors from the LTAD perspective to understand the talent development pathways followed from a holistic view (Zuber et al., 2016).

### A Holistic Approach to Career Development Model

The career transitioning model by Wylleman et al. (2004) explained athlete development pathways from a holistic perspective. Alferman and Stambulova (2017) elaborated that the holistic development model defines athletes proceeding through career stages with specific transitions apparent throughout the athletic career. The transitions athletes experience are categorized according to the degree of predictability and labeled as normative or non-normative (Wylleman et al., 2013). Normative transitions are predictable and anticipated, and include, for example, the transition of athletes from junior to senior competition, from secondary to tertiary education (Wylleman et al., 2013). In contrast, non-normative transitions are unpredictable, unanticipated, and involuntary such as a serious injury, loss of a coach or being left out of the team (Wylleman et al., 2013). For the purpose of this study, holistic athlete development as described in terms of the normative transition from junior to senior sport participation will be elaborated on. The model emphasizes factors athletes need to cope with while transitioning and progressing through their athletic careers (Wylleman et al., 2013). An athletic career span over numerous levels of competition and progress through various talent development phases (Stambulova et al., 2009). The phase descriptions are generic and may differ for subsets of specific sporting codes, genders and ages (Stambulova, 1994).

Existing athletic career or talent development pathway models (Taylor and Ogilvie, 1994; Wylleman et al., 2004; Balyi et al., 2013) emphasize phase demands, influential factors and resources needed for successful transitions and coping during different phases (Stambulova et al., 2012). Coaches and significant others play an integral part during the different phases and transitions, and therefore coaches need to be cognizant of the demands and challenges athletes face when entering a normative transition (Vallée and Bloom, 2005). Phase demands are generally described across four domains of development, such as athletic, psychological, psychosocial and academic/vocational (Wylleman et al., 2004). This study on student-athletes will focus on the athletic, psychosocial and academic/vocational levels of the holistic model of talent development during the transitioning of athletes from junior to senior sport levels.

#### Athletic Level Talent Development

Athletic-level talent development within a South African context in this study includes athletes between the ages of 18 and 25 years. During this stage, the athletes transition from junior to senior level during the progression from secondary to tertiary education (Wylleman et al., 2004). On the athletic level of the holistic model, the transition from junior to senior competitive levels refers to the athletic-level transition from the developmental to mastery stage (Wylleman et al., 2013). As such, during the developmental phase, the athlete is recognized as talented with training and competition intensifying (Wylleman et al., 2013). The athletic developmental phase ends more or less between the ages of 16 and 18 years, after which athletes transition to the mastery phase and become student-athletes as they enter tertiary institutions. Many junior athletes might be regarded as top juniors at the end of the development phase; however, when entering their first year at university as athletes, they generally have to start at the lower end in terms of athletic ability and achievement (Wylleman et al., 2013). Even though athletes are elite junior athletes, they are almost deemed rookies at senior level and have to invest more time in athletic development to compete against the more mature and experienced senior athletes. Rookie seniors (first-year students) often experience the transition from developmental to mastery phase as extremely challenging, ascribed to factors such as injuries, financial instability or insufficient support or resources (Australian Sports Commission, 2003; Wylleman et al., 2013). As a result of adjustment difficulties relating to the higher athletic level, on average, only one out of three junior athletes transitions successfully into senior competition (Australian Sports Commission, 2003; Hollings, 2013). Student-athletes find it difficult to adapt to the training environment, characterized by new coaches, venues, different and more demanding scheduling and training as well as new team members. The changes in training regime, coach and team mates are added pressure to new and unique challenges student-athletes face (Hollings, 2013). The adjustment to competition, lifestyle pressures and decrease in support systems within the immediate surroundings, reflect areas of concern for student-athletes during the transition to the mastery phase (Hollings, 2013; Wylleman et al., 2017). For student-athletes to utilize the university training environment effectively, specific guidance and assistance from university coaches and personnel are prerequisite to overcome transitional and mastery phase demands (Wylleman and Lavallee, 2004; Hollings, 2013). The transition from the developmental to mastery phase takes approximately 2 years and can only be achieved if athletes have a range of support systems and individuals to facilitate adaptation (Australian Sports Commission, 2003). Support includes aspects on athletic, academic and psychosocial level (Wylleman et al., 2013).

#### Psychosocial Level

In general, first-year students move away from home to attend university and subsequently lose the support system comprising parents, friends and coaches resulting in a dramatically changed psychosocial context (Wylleman et al., 2013, 2017). In addition to the changes on psychosocial level, student-athletes identified peers as a crucial part to sustain efforts on and off the field (Wylleman et al., 2013). Although athletes need peer support, during the mastery phase, student-athletes focus almost exclusively on their athletic career, often adversely impacting friendships, which could lead to feelings of isolation (Hollings, 2013). Student-athletes who claimed a lack in support from coaches and team personnel often do not progress during the mastery phase and discontinue participation (Hollings, 2013). Similarly, student-athletes indicated a need for sport administrators to be sensitive to their novice state as they need self-confidence to improve their athletic career (Hollings, 2013). Coaches’ feedback to student-athletes should be positive and aimed at building the athletes’ perception of social acceptance and perceived improving athletic ability (Vallée and Bloom, 2005; Hollings, 2013). Likewise, student-athletes need the psychosocial reinforcement regarding their role and position within a team and coaches deliberately need to focus on fulfilling this need (Henriksen et al., 2013). The support will assist student-athletes to cope with various social and psychosocial factors, especially since the tertiary educational environment is more demanding, and influences development pathways to success (Wylleman et al., 2013).

#### Academic/Vocational Level

Student-athletes often struggle to maintain a balance resulting in conflicting academic and training demands (NCAA, 2016). Academic workload requires student-athletes to maintain a specific grade average while athletic demands require maintenance of a high level of performance and fitness. Therefore, a supportive environment to fulfill both commitments is a necessity (Enoksen, 2002; Hollings, 2013). Coaches who provide positive and supportive feedback to students regarding their academic achievements, assist athletes to maintain training and academic achievement (Brown et al., 2015). On the contrary, coaches who do not function beyond the normal sport environment to provide academic encouragement to student-athletes, add to students’ struggle to cope academically and athletically (Hollings et al., 2014). The university talent development environment void of the necessary academic support, needs to be addressed with a specific focus on enhancing athletes’ chances of pursuing the completion of a diploma or degree as well as progressing in their athletic abilities to compete at elite levels (Sotiriadou and De Bosscher, 2013; Wylleman et al., 2017).

The focus of the research reported on underscores various influential factors, *inter alia*, athletic, psycho-social, and academic demands, experienced by athletes transitioning from secondary to tertiary education. The holistic developmental model supposes that for elite junior athletes used to the demands of competitive elite sport while at high school, with numerous hours of training, coaching regimes, competitions, and pressures, the progression from junior to senior level would be challenging (Wylleman et al., 2013). Elite junior athletes need specific guidance and support to cope with demands during and after the transition (Wylleman et al., 2013). Referring to the LTAD model, athletes following the late specialization development pathway, and transitioning from secondary to tertiary education, most often will have completed their stage 4 of training to compete, or training to win (stage 5). These stages are generally for athletes who are specializing within a primary sport and are seen as the best of the junior elite competitive level while completing secondary education (Higgs et al., 2019). This study therefore evaluated the specific context of the talent development pathway followed by student-athletes transitioning from secondary to tertiary education and the factors influencing the current university sport talent environment.

## Research Methodology

### Study Design

A descriptive research design following a single-cross-sectional approach was implemented.

### Sampling Method

Student-athletes from two universities within the Gauteng Province, South Africa comprised the sample frame from which data was collected. The sample frame was calculated according to the number of team members per sporting code, identified to be part of the study. Players from the rugby, netball, soccer, cricket, basketball, athletics, hockey, volleyball, and dance sporting codes from the two universities comprised the sample frame. However, students competing in any of the University Sport South Africa (USSA) sporting codes may have participated in the study (if they were to hear about the study). In order to gain an understanding of the talent development pathway of student-athletes, purposive sampling was used, where coaches of the identified sporting codes were contacted and asked to provide time during a training session for the researchers to recruit and administer the questionnaire voluntarily and anonymously. Coaches allowed the researcher time at the onset of a team training session at the university grounds, to inform students of the study purpose and administer the questionnaire in the presence of the coach. Three hundred and twenty questionnaires (*N* = 320) were distributed for data collection, of which 289 were returned questionnaires were returned rendering a response rate of 90%. Twenty-two questionnaires were incomplete and therefore data analysis was conducted on 267 complete questionnaires.

### Participants

The research participants consisted of a convenient sample (*n* = 267) of student-athletes (mean age = approximately 19.63 years; SD = 0.281; range = 18–33) participating at various levels of competition, for example university level (*n* = 96; 35.8%), provincially (*n* = 66; 24.9%) and nationally (*n* = 39; 14.7%). A biographical profile of the sample indicated that the gender distribution was close to equal with 50.6% (*n* = 135) of the sample male and the remaining 49.4% (*n* = 132) female. As could be expected, 91.4% of the sample representing 244 respondents were between the ages of 18 and 25 years. Only 8.6% (*n* = 23) of the sample were in the 26 to 33 years’ age category. In terms of participation duration, the majority of the sample took part in the specific sport for more than 6 years (*n* = 164; 61.7%), followed by respondents who participated for approximately a year (*n* = 31; 11.7%), those who participated for 5 years (*n* = 18; 6.8%), 6 years (*n* = 16; 6%), 4 years (*n* = 15; 5.6%), 2 years (*n* = 11; 4.1%), 3 years (*n* = 9; 3.4%) and less than a year (*n* = 2; 0.8%). With reference to the study year of respondents, 29.8% representing 79 respondents were, respectively, in their first and second year of studies, followed by respondents in their third year of study (*n* = 49; 18.5%), postgraduate students (*n* = 42; 15.8%) and respondents who selected other (fifth year or more) (*n* = 16; 6%). Categorization of student-athletes into the various developmental phases were confirmed by the researchers in accordance to years the student-athletes participated in a given sport. Student-athletes were categorized into groups, viz. novice (*n* = 44), advanced (*n* = 24), and elite athletes (*n* = 197). The categorization was in accordance with participation years in competitive sport where the novice group (16.5%) indicated that they have been competing for 0 to 2 years, advanced group (9.0%) between 3 and 4 years and the elites (74.4%) for 5 years or more.

When considering the type of sport respondents participated in, 19.5% (*n* = 52) of the sample played rugby, 17.6% (*n* = 47) participated in soccer, 11.2% (*n* = 30) netball, 2.6% (*n* = 7) played cricket, 1.9% (*n* = 5) took part in athletics, 16.5% (*n* = 44) participated in basketball, 6.7% (*n* = 18) played volleyball, 2.2% (*n* = 6) did body building, 5.2% (*n* = 14) took part in dance, while the rest of the sample (*n* = 5; 1.9%) selected other. In terms of training time, more than half the sample (*n* = 134; 50.4%) indicated training time exceeding 6 h weekly, followed by 5 h per week (*n* = 49; 18.4%), 4 h (*n* = 41; 15.4%), 3 h (*n* = 18; 6.8%), 2 h (*n* = 21; 7.9%), and 1 h a week (*n* = 3; 1.1%). Looking at the level respondents participated in the sport, 35.8% (*n* = 95) of the sample competed at university level, followed by inclusion in a provincial (*n* = 66; 24.9%) and national (*n* = 39; 14.7%) or USSA (*n* = 39; 14.7%) team. Furthermore, 1.5% of the sample participated in a World Student Team, while 7.2% (*n* = 19) categorized themselves as professional and 1.1%, representing three respondents, selected other. Sequentially, 16.5% (*n* = 44) of the sample were categorized as novices, 9.0% (*n* = 24) advanced and the majority of the sample could be categorized as elites (*n* = 198; 74.4%).

### Data Collection Procedure

Data was collected over a three-week period during which the primary researcher requested permission from university coaches to administer the questionnaire in the course of an official training session. The researcher traveled to the respective universities’ sports grounds (data collected pre-COVID) and student-athletes completed the paper-based questionnaires in the presence of the researcher. Standard ethical protocol was observed according to the institutions’ prescription at that time. Participants were informed about the purpose of the study and anonymity was ensured should they voluntarily participate. Participants were advised that they could withdraw from participation at any stage, and were assured of response confidentiality. Even though organizational permission was granted to conduct the study, participating universities requested to remain anonymous. Ethical review and approval was at the time of data collection not a requirement for the study on human participants in accordance with local legislation and institutional requirements at the time when study was conducted.

### Measuring Instrument

Primary data was gathered by administering the original Talent Development Environment Questionnaire (TDEQ) by Martindale et al. (2010) to which a section requesting biographical data was added. The biographical section included items relating to gender, age, participation duration, year of study, type of sport, weekly training time, participation level, and developmental phase. The TDEQ is a 68-item questionnaire scored on a six-point Likert scale with response categories ranging from 1 (strongly agree) to 6 (strongly disagree). To counter acquiescence 15 negatively worded items were included in the measuring instrument and reversed scored for analytical purposes (Martindale et al., 2010). The TDEQ has proven reliability and validity internationally (Martindale et al., 2010). *Per se*, the Cronbach Alpha coefficients of the TDEQ ranged from 0.616 to 0.978. Furthermore, the internal consistency for the total scale was 0.805, with seven-identified factors scoring 0.978, 0.616, 0.913, 0.730, 0.899, 0.618, and 0,618, respectively. Construct validity was computed by means of exploratory factor analysis reverting a seven-factor structure with eigenvalues ranging from 33.76 to 0.979, accounting for 64% of the total explained variance. Confirmatory factor analysis (CFA) was not computed in the study by Martindale et al. (2010). The seven factors identified included long-term development focus, quality preparation, communication, understanding the athlete, support networks, challenging, and supportive environment and long-term development fundamentals.

To validate the TDEQ for the South African context, a principal component analysis with oblique (Oblimin) rotation was computed. Principal component analysis was used as the method is deemed psychometrically sound avoiding potential factor indeterminacy associated with other approaches (Pallant, 2011). The rationale for the mentioned validation of the TDEQ measurement model were two-fold, namely as a result of a CFA not being performed in the study by Martindale et al. (2010) the number of factors were not confirmed and to avoid measurement errors in subsequent analysis. Bartlett’s test of sphericity was statistically significant (X2 = 7963.469; DF = 2278; *p* = 0.000^∗∗^), indicative of sufficient correlation between variables to substantiate exploratory factor analysis. The Kaiser-Meyer-Olkin (KMO) measure of sampling adequacy returned a value of 0.892, providing evidence that the sample size was acceptable (Jonck et al., 2018). As factor analysis can be influenced by outliers, normality tests with plots were performed to determine whether the 5% trimmed mean values differ from the mean values (Pallant, 2011). Results determined that 17 components had an eigenvalue exceeding 1, accounting for 64.73% of the total variance. Non-etheless, an inspection of the scree plot indicated a clear break after the fourth factor. To verify the number of factors, a Monte Carlo parallel analysis was performed with the Statistical Package for Social Sciences (SPSS) version 26. Results obtained from the simulation specified that four components had eigenvalues exceeding the criterion value for a randomly generated data matrix consisting of 1000 cases. Sequentially, confirmatory factor analysis was performed with a four-factor rotation. Confirmatory factor analysis was computed instead of structural equation modeling since the number of factors were predetermined by Martindale et al. (2010).

Subsequent to the confirmatory factor analysis, four underlying dimensions were identified (see Table 1). Item loadings above 0.3 were considered as the cut-off point, as suggested by Pallant (2011).

**TABLE 1:** Forced four-factor component matrix.

Questionnaire item	Component			
	1	2	3	4
Question 62	0.815			
Question 61	0.754			
Question 56	0.738			
Question 54	0.726			
Question 68	0.708			
Question 58	0.683			
Question 63	0.678			
Question 49	0.672			
Question 57	0.669			
Question 64	0.669			
Question 40	0.666			
Question 60	0.654			
Question 53	0.633			
Question 65	0.601			
Question 50	0.593			
Question 52	0.580			
Question 59	0.562			
Question 67	0.543			
Question 43	0.535			
Question 24	0.416			
Question 36	0.399			
Question 55	0.391			
Question 23	0.376			
Question 39	0.349			
Question 42	0.338			
Question 11	0.328			
Question 3	0.312			
Question 31		0.739		
Question 25		0.655		
Question 32		0.642		
Question 33		0.636		
Question 19		0.578		
Question 35		0.576		
Question 27		0.574		
Question 13		0.544		
Question 6		0.478		
Question 17		0.466		
Question 9		0.463		
Question 4		0.421		
Question 51		0.417		
Question 47		0.388		
Question 30			0.727	
Question 48			0.690	
Question 44			0.656	
Question 29			0.535	
Question 41			0.528	
Question 28			0.524	
Question 18			0.501	
Question 15			0.477	
Question 38			0.467	
Question 37			0.447	
Question 66			0.444	
Question 14			0.434	
Question 22			−0.363	
Question 34			0.349	
Question 20			0.341	
Question 26			0.337	
Question 12				−0.470
Question 10				−0.444
Question 16				−0.441
Question 7				−0.434
Question 1				−0.324
Question 8				−0.324

*Factor 1 incorporated items relating to a supportive and challenging environment with factor loadings ranging from 0.815 to 0.312. Examples included emotional support to develop confidence (factor loading 0.815), support to engage in experiential learning (factor loading 0.754) and supporting influence (factor loading 0.738). Factor 2 underscored development fundamentals with factor loadings ranging between 0.739 and 0.388. Specific items included sports guidance (factor loading 0.739), mentorship (factor loading 0.655) and fortitude development (factor loading 0.642). Factor 3 focused on support networks with factor loadings ranging from 0.727 to 0.337. Specific items focused on developmental networks such as physiotherapist, sport psychologist, strength trainer, and nutritionist, to mention a few (factor loading 0.727), communication between coach and developmental network (factor loading 0.690), communication between coach and parents (factor loading 0.656) and quality preparation (factor loading 0.535). The last factor emphasized a long-term development focus with specific topics covered including motivation, SWOT (strength, weaknesses, opportunities, and treats) analysis, goal setting and managing peer group influence. Based on the results presented, 5 items were excluded from further analyses and only a four-factor structure were retained contrary to the seven factors suggested by Martindale et al. (2010). Hence, the measurement model was adapted to avoid measurement error in subsequent statistical analyses.*

Within the current sample, the TDEQ reported a Cronbach alpha coefficient of 0.94 for the total scale. All the sub-scales produced more than acceptable reliability estimates ranging from 0.805 to 0.941. Specifically, the supportive and challenging sub-scale had an alpha coefficient of 0.941, development fundamentals reverted the same at 0.824, support network reverted an alpha coefficient of 0.840 and long-term development focus had an internal consistency of 0.805.

### Data Analysis

The Statistical Package for Social Sciences (SPSS) Version 26 was utilized to perform descriptive and inferential statistical analyses. Principal factor analysis and CFA based on a Monte Carlo parallel simulation (*per se* randomly generated data matrix with 1000 cases) were performed to validate the measuring instrument and to identify statistical significant dependent variables. Cronbach’s alpha coefficient was used to provide evidence for reliability. Inferentially, multivariate analysis of variance (MANOVA) was used to determine the influence of developmental phase on TDEQ components. Hierarchical multiple regression was computed controlling for developmental phase to construct a statistical model determining the individual contribution of each component in order to investigate the developmental pathway. Statistical significance (*p*-value) was set at either 0.01 or 0.05. Practical significance was set at the recommended levels of 0.2 (small effect), 0.5 (medium effect), and 0.8 (large effect) [see Kraemer et al. (2003)].

## Results

Results will be presented in two sections, namely descriptive and inferential statistical results.

### Descriptive Statistical Results

In light of the developmental classifications elucidated in the methodology section, it is prudent to consider respondents’ year of study at university to gain insight into the duration student-athletes within the different talent developmental pathway categories have been competing in competitive sport after transitioning into tertiary education and the implications considering the two theoretical models. Table 2 presents a cross-tabulation indicating the developmental categories according to year of study.

**TABLE 2:** Cross-tabulation of developmental categories according to year of study.

Talent developmental pathway category		Year of study					
		1	2	3	Post-graduate (4+)	Other	Total
Novice	*N*	17	18	5	2	1	43
	%	21.5	22.8	10.2	4.9	6.3	100
	Total%	6.4	6.8	1.9	0.8	0.4	16.3
Advanced	*N*	7	5	9	2	1	24
	%	8.9	6.3	18.4	4.9	6.3	9.1
	Total%	2.7	1.9	3.4	0.8	0.4	9.1
Elites	*N*	55	56	35	37	14	197
	%	69.9	70.9	71.4	90.2	87.5	74.6
	Total%	20.8	21.2	13.3	14.0	5.3	74.6
Total	*N*	79	79	49	41	16	264
	%	100	100	100	100	100	100
	Total%	29.9	29.9	18.6	15.5	6.1	100

According to Table 2 above, 16.3% (*n* = 43) of the sample could be deemed novice student-athletes who started competing in a new sport while at university or participated in a sport before entering university, but not at a competitive level (first years *n* = 17, 6.4%; second years *n* = 18, 6.8%; third years n = 5, 1.9%; fourth year or more *n* = 3, 1.2%). The advanced student-athlete development pathway group (total *n* = 24, 9.1%; first years *n* = 7, 2.7%; second years; *n* = 5, 1.9%; third years *n* = 9, 3.4% and fourth year or more *n* = 3, 1.2%), indicated that they have been competing for 3 to 4 years competitively. The elite student-athlete talent development group consisting out of first year student-athletes (*n* = 55, 20.8%), second years (*n* = 56, 21.2%), third years (*n* = 35, 13.3%), and fourth year or more (*n* = 51, 19.3%) had more than 5 years’ experience in competitive junior sport.

Descriptive statistics, including measure of central tendency are displayed in Table 3 below.

**TABLE 3:** Descriptive statistics for TDEQ variables.

Variable	Mean	Median	SD	Minimum	Maximum
SCE	62.33	60.00	20.008	27.00	130.00
DF	48.95	49.00	11.856	14.00	76.00
SN	46.91	46.00	12.135	21.00	87.00
LTDF	16.09	15.00	6.006	7.00	39.00

*SD, standard deviation; SCE, supportive and challenging environment;*

*DF, development fundamentals; SN, support network; LTDF, Long-term development focus.*

*Technical: scoring was done on a continuum from positive to negative with the median representing 50% or the midpoint.*

As can be seen from Table 3, the mean score reported for supportive and challenging environment (mean = 62.33; median = 60.00) suggests that respondents in the current sample displayed a slightly negative stance toward the talent development environment. Similarly, support networks also reverted a slightly negative response (mean = 46.91; median = 46.00), as was the case with long-term development focus (mean = 16.09; median = 15.00) and development fundamentals (mean = 48.95; median = 49.00).

### Inferential Statistical Results

As a pre-requisite for the hierarchical regression modeling, a Pearson Product-moment correlation was computed to determine whether the TDEQ facets were statistically significantly correlated (see Table 4).

**TABLE 4:** Pearson Moment Correlation results for TDEQ facets.

		SCE	DF	SN	LTDF
SCE	R	1			
	P				
DF	R	0.028	1		
	P	0,651			
SN	R	0.677	−0.142	1	
	P	0.000**	0.020*		
LTDF	R	0.664	−0.005	0.570	1
	P	0.000**	0.938	0.000**	

*SCE, supportive and challenging environment; DF, development fundamentals; SN, support network; LTDF, Long-term development focus.*

Technical: *p*-values are reported on in the table and the asterisk next to the *p*-value is indicative of the level of significance set at either *p ≤ 0.05; **p ≤ 0.01.

*Technical: strength of the *R*-value is interpreted as small effect (r = 0.10 to r = 0.29), medium effect (r = 0.30 to r = 0.49), and large effect (r = 0.50 to r = 1.00) see Pallant (2011).*

The correlation analysis revealed a statistically significant correlation on the 99th percentile between support network and support and challenging environment as indicated by the *R*-value of 0.677 and a *p*-value of 0.000^∗∗^. The strength of the relationship is large and as a two-tailed analysis was performed the correlation is positive. Thus as there is an increase in support network there will be a concomitant increase in support and challenging environment. Furthermore, there was a statistically significant negative correlation between support network and developmental fundamentals as seen from an *R*-value of −0.142 and a *p*-value of 0.02^∗^. The strength of the correlation is small and there is an inverse direction. Thus as there is an increase in support network there was a decrease in developmental fundamentals. Long-term development focus reverted statistically significant correlations with both support and challenging environment (r = 0.664; *p* = 0.000^∗∗^) and support networks (r = 0.570; *p* = 0.000^∗∗^). The effect size in both instances were strong and positive indicative of an increase in one variable will result in an increase in the other.

MANOVA was used to determine whether the developmental phase statistically significantly influences the dependent variables viz. supportive and challenging environment, developmental fundamentals, support networks and long-term development focus. The results are illustrated in Table 4.

As can be deduced from Table 5 above, the various developmental categories did not statistically significantly differ in terms of the four components of talent development environment within the South African university sport context indicative of a hypothesized singular approach for the different developmental categories. In terms of practical significance, all the dependent variables reported a small effect as deduced from the partial eta squared values of less than 1 hence warranting future research.

**TABLE 5:** Multivariate analysis of variance results for developmental phase and aspects related to talent development environment.

Independent variable	Dependent variable	*F*	*df*	*P*	*Partial eta squared*
Talent-development pathway categories	Supportive and challenging environment	2.133	2	0.121	0.016
	Development fundamentals	0.890	2	0.412	0.007
	Support networks	1.102	2	0.334	0.008
	Long-term development focus	1.750	2	0.176	0.013

df, degrees of freedom; p, statistical significance; partial eta squared, practical significance.

*p ≤ 0.05; **p ≤ 0.01.

Hierarchical multiple regression was performed to investigate the development pathway controlling for developmental phase. The results are displayed in Table 5.

Developmental phase together with supportive and challenging environment were entered into model 1, explaining 1.1% of the variance in developmental pathway (f = 2.818; *p* = 0.094) (see Table 6). After entry of development fundamentals in model 2, the total variance explained by the model was 1.7% (f = 2.230; *p* = 0.110). The additional contribution of developmental fundamentals was 0.6%. In model 3, support networks were entered into the equation with no change to the model (f = 1,481; *p* = 0.220). In model 4, long-term developmental focus was entered. The model in totality explained 2% of the variance with the last mentioned contributing 0.03% to the model (f = 1.324; *p* = 0.262). None of the factors statistically significantly predicted the talent development pathway for the specific South African university athlete context. The South African context differs vastly in comparison to the same internationally. Ascribed to South Africa’s segregation history youths from rural areas are disadvantaged in terms of participation in and access to quality sports development opportunities (Motlhaolwa, 2016). Therefore, the talent development environment, the factors indicative thereof and the measuring instrument should be context specific. What is more, a limitation reported by Martindale et al. (2010) was the generic-nature of the measuring instrument. In accordance with the identified limitation the authors suggested the development of a context-specific measuring instrument as explicit needs intrinsic to the environment influence talent development (Martindale et al., 2010).

**TABLE 6:** Hierarchical regression analysis results controlling for developmental phase.

Model	R	R^2^	Adjusted R^2^	R^2^ change	*p*
1	0.103	0.011	0.007	0.011	0.094
2	0.129	0.017	0.009	0.006	0.202
3	0.129	0.017	0.005	0.000	0.998
4	0.141	0.020	0.005	0.003	0.356

*p ≤ 0.05; **p ≤ 0.01.

## Discussion

The cross-tabulation results indicate that a total of 43 student-athletes started competing in a new sport while at university or they participated in a sport before entering university, but not at a very competitive level. Considering the LATD phases, student-athletes entering university as first-year students should have completed stage 4, *learning to compete* as well as stage 5 of *training to compete*, between the ages of 15/16 and 19/20 years (females and males, respectively) (Balyi et al., 2013). During these stages, athletes are supposed to optimize individual and position-specific skills, aimed at maximizing performance at junior elite level (Balyi and Hamilton, 2010). As first- and second-year students classified into the novice developmental pathway category (*n* = 35; 13.2%), presumably these athletes were not exposed to previous high-level training and competitive practice. Novice development pathway student-athletes will be lagging behind in technical, tactical, physical and mental skills development (Higgs et al., 2019), since indicating participation in competitive sport for less than 2 years. This implies that, as first- and second-year students, might not have received specialized coaching and training while attending secondary school, as is necessary during the learn to compete and train to compete stages. The lower skills development and lack of high level training and competitive participation while in high school may be ascribed to a lack in resources, available coaches and competition opportunities (Fraser-Thomas and Côté, 2009).

According to the holistic perspective by Wylleman et al. (2013), novice student-athletes can be considered to still fall within the athletic development phase aimed at enhancing skills. This implies that novice student-athletes are in need of more training and skills development provided by university coaches to overcome the deficit in skills development and competition exposure not received before the transition into tertiary education sport (Hollings et al., 2014). The transition from junior- to senior-level sport already poses significant problems, even for the elite and well-trained junior athlete who has competed for many years while still at high school (Wylleman et al., 2013). The transition of an untrained, or lower-level trained athlete into tertiary education characterized by a higher competitive level, will pose greater athletic demands on a novice athlete. At the athletic development level, university coaches and additional staff will therefore have to provide added training, instruction and support to the novice student-athletes, since they are behind in the LTAD and holistic view development pathway. Novice student athletes do not have the training and competitive elite sport participation experience, and need to adapt to the enhanced training regimes and time allocated, higher academic workload, new competitive league participation and perform at the higher competitive levels (Hollings, 2013). Coach instruction on technical, tactical, physical, mental and competitive skills will have to be a primary focus to these novice student-athletes (Higgs et al., 2019). On the psychococial level, novices will need to establish a close relationship with their new coach, team mates and class members in order to cope with the changes in being away from home as well as the pressures of training and competing (Wylleman et al., 2013; Higgs et al., 2019).

Considering that the novice student-athlete category comprises 16.3% of the total group, the coaches are posed with practical challenges. In this regard, usually, where university coaches are expected to focus attention on attaining peak physical, technical, tactical and mental performance to maximize outputs at senior elite competitive level, they will have to spend a great deal of time to train and develop a small group of novice student-athletes’ skills to compensate for a lack in skills development within the team set-up. Even though it is commendable that students are willing to increase their sport participation and strive to engage at higher competitive levels when at university, the novice developmental category athlete is not up to the athletic standard needed at tertiary level and may influence the talent development environment of the particular university sport. The novice athlete may develop and progress during his/her university participation years, since newly attained exposure to skilled coaches, better training environments, competition opportunities, facilities and funding will facilitate development (NCAA, 2016). However, with an athletic skills development deficit, coaches will have to revert back to learning to compete skills development phase, compared to the age appropriate development phase where the focus is on training to compete skills enhancement.

The advanced student-athlete development pathway group (*n* = 24; 9.1%), indicated that they have been competing for 3 to 4 years competitively. This means that the first- and second-year students (*n* = 12) could have completed the *learning to compete* (LTAD stage 4) during their secondary education years, and have already developed sport-specific skills, maximized fitness and competed at higher junior competitive levels, before transitioning into university (Balyi et al., 2013). These student-athletes are then ready to enter the *training to compete* (LTAD stage 5) stage as they transition into tertiary education. The total group of the advanced student-athlete category may possess more technical and physical skills compared to the novice category, since they have been competing a few more years within the specific sport. The advanced category group may also be able to cope better with the enhanced training routines, higher training hours, academic workload and competitive levels, since they already had exposure during secondary school within elite junior levels (stages 4 and 5 of LTAD and the athletic developmental stage). Even though the advanced development group of student-athletes may have more experience and athletically increased development compared to the novice group, coaches will still need to spend a great deal of time to progress their athletic level from development to mastery stages for sport-specific physical, technical, tactical and mental skills according to the transitional model by Wylleman et al. (2013). The advanced student-athlete group will experience the normative transitional demands on athletic, psycho-social and academic levels and will be in need of coach, additional staff and peer support to make a successful transition.

Comparing the elite student-athlete talent development group (*n* = 197; 74.6%) with the novice and advanced categories, it is evident that a large number of student-athletes transitioning into university sport have had more than 5 years’ experience in competitive junior sport. A total of *n* = 111 first- and second-year students in the elite category comprises 42% of the total student-athlete group with 5 years or more experience in competitive sport participation. The division of students into this category coincides with the athletes progressing through LTAD stages 4 and 5 at the desired ages (15–23 years), and are therefore ready to transition into tertiary sport according the model by Wylleman et al. (2004). Adding third-year students (*n* = 35) to first- and second-years, the elite category students totals 147 students (55%) in the total group, with 5 years or more experience. These athletes are likely to be closest to a standard late specializing talent development pathway. Elite group athletes possibly would be at a higher technical and physical developmental level, and coaches would be able to engage them at a higher level regarding skills development and mastery for the achievement of senior level success. This is indicative of the correct talent development pathway followed according to the prescribed ages according to LTAD and holistic development pathway models (Wylleman et al., 2004; Balyi and Hamilton, 2010). Considering that half of the student-athlete group possesses the necessary training and competitive experience when entering university sport, coaches and support staff will have to diversify their training goals and sessions to accommodate the lack in skills development for the different skill levels, specifically accommodating the novice and advanced athletes. Likewise, coaches need to invest a considerable amount of effort to help students cope with the higher training and competitive levels, as they transition from junior to senior elite competitive levels. Considering that half of the students have not developed according to the LTAD and holistic development model, and therefore did not follow a late development pathway, coaches have a diversified skill level group of students to work with. The results further indicate that the fourth years’ and older students (*n* = 51), also had the opportunity to continuously train and compete at high competitive levels while at university, and a small number of them may have done so before they transitioned into tertiary education. The elite category student-athletes (*n* = 197) would be used to higher levels of training, physical conditioning, sport- and position-specific instruction, as well as accustomed to the pressures of higher levels of competition. In this regard, the coaches and staff will have to play a supporting role to facilitate the transition from elite junior to elite senior levels (Hollings et al., 2014) and from the learning to compete to training to compete phases. The elite student-athletes would be at a higher experience-, physical-, mental- and skills level compared to the other two categories of students. Considering that the transition from junior to senior elite competitive levels is already tricky and poses numerous challenges, even for elite junior athletes who have more or less 4 to 5 years’ competitive training and competing experience (Wylleman et al., 2004), the more so the challenges would be for the novice and advanced category student-athletes who transitioned into tertiary education and sport with less training, skills development and experience in elite competition.

The differences in years’ experience and exposure to competitive junior and senior elite sport were highlighted through the results in Table 2. The categorization of the three talent developmental pathways for student-athletes indicates that student-athletes progressed through the late specializing talent pathways at different rates and ages as juniors. This resulted in athletes entering university sport with varying skill levels, which poses numerous challenges during the transition and different skills levels of the student-athlete group. The different level athletes will continue to progress through the talent development pathways of *training to compete* and *training to win* during their tertiary education. In order to determine the factors influencing the university talent development environment for the varying pathway category student-athletes, the Talent Development Environment Questionnaire was used as measuring instrument.

Results pertaining to the factors influencing the talent development environment, namely *supportive and challenging environment*, *developmental fundamentals*, *support networks*, and *long-term development focus* associated with the change from junior to senior competitive levels, coincide with the transition from secondary to tertiary education, and corresponds with previous research findings (Segers et al., 2011; Mills et al., 2014a).

The first identified factor, *supportive and challenging environment*, encompasses the support student-athletes require during and after transitioning. Primarily, student-athletes need emotional and psychological support within the new challenging and competitive athletic environment. The support also extends wider to include helping respondents cope with the increased academic workload, and adapting to psychococial changes regarding significant other relationships (Wylleman et al., 2017; Reverberi et al., 2020). Student-athletes must balance the new challenging environment on athletic, psycho-social and academic levels with a strong supportive influence from the coach and numerous support services to advance and facilitate the development of various skills (Van Puyenbroeck et al., 2018). In this regard, a supportive and continuously challenging environment positively influences athletes’ conflicting demands experienced, if dealt with in a constructive manner through proper guidance from coaches and support staff (Aalberg and Sæther, 2016; Reverberi et al., 2020). The low mean score for the supportive and challenging environment factor (Table 3) is indicative of student-athletes experiencing a low level of support to cope with the transition and demands associated on various levels. Just less than half (45%) of the student-athletes are categorized as novice or advanced pathway category athletes, with lower levels of specialized training and competition level experienced when entering senior elite university sport. The transition from junior to senior elite is already demanding on elite athletes, where the novice senior athlete will experience higher demands to cope, ascribed to the existing skills gap. The statistically significant correlation on the 95th percentile between supportive and challenging environment and development fundamentals, indicates the close relationship between a wide spectrum of support provided to develop certain fundamental aspects for holistic athletic development.

The second factor, *developmental fundamentals*, refers to the guidance provided by coaches and support staff, which plays an instrumental role in the development of the fundamental skills for enhanced athletic performance (Aalberg and Sæther, 2016). Therefore, coaches not only need to develop the athletic abilities of student-athletes regardless of their developmental pathway, but also provide guidance and mentorship to ensure systematic progression longitudinally emphasizing mental and physical development (Poole, 2016). The quality of developmental fundamentals and a structured approach within the talent environment has a direct influence on athletic success and achievement at senior elite competitive levels (Burnett, 2017). Therefore, coaches are instrumental in guiding student-athletes to incorporate specific fundamental aspects such as physical conditioning, mental preparation, technical and tactical skill acquisition and overall performance enhancement (Van Puyenbroeck et al., 2018). The low mean score for the developmental fundamentals factor (Table 3) indicates that students do not perceive the coach to provide adequate guidance and structure to address the overall athletic development needed for the progression of their sporting career. The results further indicate that, within this South African university context, coaches have a group of student-athletes with varying developmental levels, and therefore may struggle to design and incorporate long-term, high-level training sessions, aimed at enhancing fundamental aspects to develop elite student-athletes.

The third factor, *support networks*, elaborates on developmental fundamentals, where the coach has the responsibility to manage and control all support networks and resources available to student-athletes at universities (NCAA, 2016). Support systems and networks at tertiary institutions refer to career guidance, academic support, personal development and athletic support (Gomez et al., 2018), while sport-specific personnel such as physiotherapists, physical conditioners, sport psychologists, medical personnel and video analysts, additionally enhance athletic performance (Hollings et al., 2014; NCAA, 2016). The negative experience by student-athletes regarding available support networks within the current university context (mean score Table 3), points to resources and support personnel unavailability, or that the coach is perceived not to manage the service in such a manner that enhances talent development. In this regard, Ogunniyi (2015) posits that a lack of proper support services hinders athletic development and is prevalent at numerous South African organizations. The outlined support services are requisite for on- and off the field student-athlete performance, since a well-balanced approach is needed to overcome the difficulties of maintaining a competitive training regime and adapting to academic and psycho-social demands (Gaston-Gayles and Baker, 2015). The support services assist the transition from junior to senior sport performance, as well as the talent development while at university (Rice et al., 2016), and further investigation into the negative perception of the support services of the current student-athlete group is warranted.

The fourth factor, *long-term development focus*, identified in this study, is supported by the literature where Poole (2016) posits that a long-term focus is a prerequisite for success. In this regard, coaches provide the long-term vision and develop periodized training and competitive cycles on macro- and meso-level, with the aim to attain the highest level of success planned over a three- to four-year period (Bompa and Buzzichelli, 2019). Since the current group of student-athletes is categorized into different talent development category pathways, the coaches’ overall long-term vision and development planning for athletes may have to be varied. University coaches may have a diversified approach to specific training objectives to address the different skills development levels of student-athletes. Varying talent development category student-athletes within a single athletic group pose specific challenges for coaches. In this regard, a coach may focus on the short term to enhance the technical and tactical skill level of the novice student-athletes, while addressing the skill development of the advanced and elite level athletes within a single group. Challenges reach beyond providing diversified training opportunities aimed at individual athletes with varying physical and technical skills levels, but also stretch beyond adequate support, guidance and facilitation of needed resources. The diversified development categories hinder coaches’ long-term planning for individual and team development (Coutinho et al., 2016). In this regard, the current South African university team consists of novice, advance and elite athletes; the coach more or less has 4 to 5 years with the student-athlete to develop individuals to the highest competitive level. The highest competitive level that will be attained by a first-year novice athlete will be much lower than an elite junior with more than 5 years’ competitive junior competition experience, before transitioning into university sport. The coach will need to carefully plan for novice, advanced and elite student-athletes to develop and enhance their skills over the long-term period. The negative responses of the student-athletes regarding the long-term development focus factor (Table 3), may be indicative of coaches not being able to design athletic development pathways to facilitate the different development categories. The statistically significant correlation on the 99th percentile between long-term development focus and developmental fundamentals is substantiated by previous findings, where various approaches and dynamic models of athlete and talent development over a long period provide insights into best practices (Coutinho et al., 2016). The nationally developed long-term participant development framework (South African Sport for Life) advances the implementation of a comprehensive system and talent environment for long-term athlete development (Burnett, 2017).

## Conclusion

The student-athlete group within the South African university context has diverse development pathways as they transition from secondary to tertiary education. Almost half of the sample followed a normal late specialization pathway and completed the LTAD learning to compete stage and entered university with adequate skills and exposure to competitive junior sport. The remaining half of the participants followed an even later specialization development pathway, where students did not receive adequate training, development and competition exposure while in the learning to compete stage. The differentiation in skills and competitive participation levels of athletes as they enter university, create practical complications for coaches to manage the talent environment. Coaches may find it difficult to support the varied developmental levels of students as they transition into university sport and this is evident from the low satisfaction of student-athletes in terms of inadequate supportive and challenging environment, fundamental development aspects, support networks and long-term development focus.

The TDEQ used to measure the talent environment created and managed by university coaches rendered four important factors, however, indicated that students did not perceive coaches to perform the functions effectively. The low model fit indicates that the four factors do not statistically significantly predict talent development pathways for the student-athletes within the various developmental pathways. The four factors are indicative of supportive actions, services and behavior from coaches and are therefore quite homogeneous. In this regard, the roles of the coaches within the development of student-athletes from diversified pathways are focused on support provided, which unfortunately seems to be insufficient. In contrast, a well-developed talent environment addresses a variety of aspects such as physical, mental, academic, psycho-social and sport-specific elements that enhance talent development. The low model fit indicates that the TDEQ is not comprehensive enough to measure the required talent development aspects for the South African student-athlete context. A different measuring instrument should be developed, context specifically, to ascertain the talent environment factors statistically significantly influencing the different developmental pathways of student-athletes.

### Limitations and Future Research

An acknowledged caveat in the research reported on subsume the use of non-probability sampling which could adversely influence the external validity of the results reported. Another caveat is that an international measuring instrument was used and had to be adapted to the South African context. It is recommended that future research be expanded to include a random sample of youth sport-athletes from secondary and tertiary educational institutions in all nine South African provinces. Furthermore, it is recommended that the influence of culture on youth sports development in the South African context be investigated. Future research endeavors could also underscore the development and validation of a South African TDEQ.

## Data Availability Statement

The raw data supporting the conclusions of this article will be made available by the authors, without undue reservation.

## Ethics Statement

Ethical review and approval was not required for the study on human participants in accordance with local legislation and institutional requirements at the time when the study was conducted. The patients/participants provided their written informed consent to participate in this study.

## Author Contributions

All authors listed have made a substantial, direct and intellectual contribution to the work, and approved it for publication.

## Conflict of Interest

The authors declare that the research was conducted in the absence of any commercial or financial relationships that could be construed as a potential conflict of interest.

## Publisher’s Note

All claims expressed in this article are solely those of the authors and do not necessarily represent those of their affiliated organizations, or those of the publisher, the editors and the reviewers. Any product that may be evaluated in this article, or claim that may be made by its manufacturer, is not guaranteed or endorsed by the publisher.
